# Closed-Loop Acoustic Stimulation During Sleep in Children With Epilepsy: A Hypothesis-Driven Novel Approach to Interact With Spike-Wave Activity and Pilot Data Assessing Feasibility

**DOI:** 10.3389/fnhum.2019.00166

**Published:** 2019-05-21

**Authors:** Sara Fattinger, Bigna Bölsterli Heinzle, Georgia Ramantani, Lucia Abela, Bernhard Schmitt, Reto Huber

**Affiliations:** ^1^Children’s Research Center, University Children’s Hospital Zurich, Zurich, Switzerland; ^2^Child Development Center, University Children’s Hospital Zurich, Zurich, Switzerland; ^3^Division of Clinical Neurophysiology, University Children’s Hospital Zurich, Zurich, Switzerland; ^4^Department of Child and Adolescent Psychiatry and Psychotherapy, Psychiatric Hospital, University of Zurich, Zurich, Switzerland

**Keywords:** high-density EEG, neurostimulation, interictal activity, electrical status epilepticus during slow wave sleep, development

## Abstract

Slow waves, the electroencephalographic (EEG) hallmark of deep sleep, can be systematically manipulated by acoustic stimulation: stimulation time-locked to the down phase of slow waves reduces, whereas stimulation time-locked to the up phase increases slow waves. Spike-waves during sleep seem to be related to slow waves, raising the question of whether spike-waves can be systematically influenced by such acoustic stimulation. In five pediatric patients, all-night EEG was recorded, combined with real-time slow wave detection. Throughout the night, acoustic stimulation was performed in a 3 × 5-min-block design (no stimulation—stimulation—no stimulation). Tones were applied time-locked either to the up or to the down phase of the detected slow waves in an alternating pattern. All patients tolerated the acoustic stimulation during sleep well. They showed high sleep quality and no signs of clinical or non-convulsive electrographic seizures. Our preliminary analysis shows no systematic effect of acoustic stimulation on spike-wave activity. Moreover, with our stimulation approach tones were distributed over a rather broad phase-range during the DOWN or UP stimulation and showed inter-individual differences in their distribution. In this study, we applied for the first time an acoustic closed-loop slow wave stimulation tool for a non-invasive manipulation of spike-wave activity. Thus, our pilot data show that closed-loop acoustic stimulation is feasible and well tolerated in children with spike wave activity during sleep. Improved precision in phase targeting and personalized stimulation parameters in a larger sample of subjects might be needed to show systematic effects.

## Introduction

Sleep has obvious effects on the electroencephalographic (EEG) characteristics of epilepsy. In particular, non-rapid eye movement sleep (NREMS) facilitates interictal epileptiform discharges (i.e., spike-wave activity), showing increased and more widespread interictal spiking compared to REM sleep (REMS) and waking (Sammaritano et al., 1991; Malow et al., 1998). A recent study with patients who underwent combined scalp and intracerebral EEG recordings revealed that this facilitation of spike-wave activity during NREMS is mainly mediated by high amplitude slow waves (Frauscher et al., 2015). Sleep slow waves are the EEG hallmark of deep sleep (i.e., NREMS stage 3) and represent a highly synchronized oscillation of the membrane potential of cortical neurons between two different voltage levels. These include a depolarized active on-state and a hyperpolarized silent off-state resulting in an alternating neuronal activity pattern, consisting of periods with neuronal activity and periods of complete neuronal silence (Steriade et al., 1993, 2001). In the scalp EEG, this activity pattern of high spatial and temporal neuronal synchronization is reflected in high amplitude slow waves with the up-phase of the slow waves corresponding to the active on-states and the down-phase to the silent off-states (Vyazovskiy et al., 2009; Frauscher et al., 2015). It has been suggested that this synchronization of neuronal activity during physiological slow waves may turn into spike-wave activity under pathological conditions (Steriade and Amzica, 1994; Steriade et al., 1994). However, evidence for this shared underlying mechanism is limited to animal experiments.

Only recently, it has been experimentally shown in healthy humans that these slow waves can be systematically manipulated by acoustic stimulation depending on the exact timing of tone onsets relative to the slow wave phase. On the one hand, stimulation time-locked to the active up-phase (i.e., the on-state, when neuronal populations are depolarized) enhances slow wave activity (SWA; Ngo et al., 2013), conceivably by facilitating a fast and efficient synchronization of neuronal activity, resulting in high amplitude slow waves (Bellesi et al., 2014). On the other hand, acoustic stimuli presented time-locked to the down-phase (i.e., the off-state, when neurons are hyperpolarized) reduces SWA (Fattinger et al., 2017). Such stimulation during the down-phase may provoke local neuronal firing (Sachdev et al., 2004; Vyazovskiy et al., 2012), which likely results in a local termination of the off-state of the ongoing slow oscillation and thereby reduces the level of neuronal network synchronization, thus reducing SWA (Fattinger et al., 2017). Importantly, the studies described above could show that such non-invasive manipulation of physiological slow waves by acoustic stimulation has direct behavioral consequences: Increasing SWA improved sleep-dependent memory consolidation (Ngo et al., 2013), whereas reducing SWA interfered with the sleep-dependent renormalization of learning capacity (Fattinger et al., 2017).

Interictal spike-wave activity in the EEG is frequent in epileptic patients and is even detected in 1%–5% of the general population (Okubo et al., 1994; Kasteleijn-Nolst Trenité and Vermeiren, 2005; Sánchez Fernández et al., 2015). Such spike-wave activity has been related to cognitive impairments, even in subjects without any clinical symptoms of epilepsy (Kasteleijn-Nolst Trenité and Vermeiren, 2005). Moreover, in epileptic encephalopathies (Scheffer et al., 2017), such as electrical status epilepticus during slow wave sleep (ESES), spike-wave activity is supposed to actively contribute to a disturbance in cerebral functioning and normal physiological development (Holmes and Lenck-Santini, 2006; Tassinari and Rubboli, 2006; Liukkonen et al., 2010). Considering that interictal spike-wave activity during sleep is related to sleep slow waves and to their underlying level of neuronal synchronization, the question arises whether such spike-wave activity can also be systematically modulated by acoustic stimulation during sleep. If so, such closed-loop acoustic stimulation may offer a novel, non-invasive tool to gain more insights into electrophysiological mechanisms of spike-wave generation during sleep and may even allow developing new treatment strategies beyond pharmacological interventions.

To test this hypothesis, we performed a prospective pilot study including five children (age range 6.9–12.1 years, two females) diagnosed with childhood epilepsies characterized by activation of spike-wave activity during slow-wave-sleep. We aimed at investigating: (1) whether acoustic stimulation during sleep is feasible in childhood epilepsy patients; and (2) whether the spike-wave activity can be systematically influenced during sleep by acoustic stimulation time-locked to the up- and down- phase of slow waves.

## Materials and Methods

### Subjects

Children diagnosed with benign epilepsy with centro-temporal spikes (BECTS), electrical status epilepticus during slow wave sleep (ESES), or generalized spike-waves in sleep, which underwent regular electrophysiological work-up between November 2015 and August 2016, were recruited from the neuropediatric outpatient clinic of the University Children’s Hospital Zurich, Switzerland. All patients fulfilled the following criteria:

Inclusion criteria:

1. Patients with BECTS
  − Age 4–12 years
  − Focal spike waves in routine EEG
  − Routine EEG within 6 months before the study night
  − a seizure history compatible with BECTS, normal development and no neurological abnormalities (an MRI was not mandatory if the electroclinical diagnosis was clear)
2. Patients with ESES
  − Age 4–12 years
  − Overnight EEG within 3 months before the expected study night
  − Bilateral synchronous high amplitude spike waves in NREM sleep
  − Spike-wave index (SWI; according to Aeby et al., 2005) between 50% and 80% during the first 30 min of NREM sleep (Scheltens-de Boer, 2009).
3. Patients with generalized spike-waves in sleep (probably genetic generalized epilepsies)
  − Age 4–12 years
  − Sleep EEG within 3 months before study night
  − Generalized spike waves in a sleep EEG

Exclusion criteria:

− Acute or progressive neurological disease
− Other clinically significant concomitant disorders
− Diffuse multifocal or generalized spike waves in sleep
− Generalized or focal motor seizure frequency >1/week (no exclusion if absence seizures)
− History of convulsive status epilepticus
− Generalized or focal motor seizures within 24 h before the study night (no exclusion if absence seizures)
− Known risk of seizures provoked by sleep deprivation
− Sleep apnea, restless legs syndrome or other severe sleep problems (parasomnia was no exclusion criterion)
− Treatment with corticosteroids, immunosuppressive drugs or vagus nerve stimulation
− Inability to follow the procedures of the study

In total, seven patients (four with BECTS, two with ESES, one with generalized spike waves in sleep) met the above criteria and were enrolled in the study. Two patients were excluded from the analysis: for the first patient (BECTS), a different study protocol (i.e., block duration of 15 min, for details see below) was applied that was adapted afterward, and another patient (BECTS) was not able to fall asleep and the recording was terminated after 4 h. This led to five patients (age range 6.9–12.1 years, two females), which were included for further analysis (for an overview, see Table 1). Prior to participation, written informed consent was obtained from all children and their parents, after providing a detailed description of the study design and aims. All recordings were performed in the sleep laboratory of the University Children’s Hospital in Zurich, Switzerland, and the study protocol was approved by the local ethics committee (*Kantonale Ethikkommission Zurich*, *Switzerland, #2015-0371*) and was performed according to the Declaration of Helsinki.

**Table 1:** Patient description.

Subject Id	Age	Sex	Epilepsy	Focus	Target Ch	Mean SWI (%)	Med
G	10.9	Male	Absence epilepsy	-	FZ	2.4	Levetiracetam (35 mg/kg)
B1	9.5	Male	BECTS	C4	C4	71.2	-
B2	12.1	Female	BECTS	F7	F7	24.2	Oxcarbazepine (30 mg/kg)
E1	6.9	Female	ESES	O1/O2	O1	69.9	Carbamazepine (22 mg/kg)
E2	9.5	Male	ESES	F3/T4	F3	70.3	Ethosuximide (23 mg/kg)

*Overview of the patients. Clinical and electrophysiological characteristics of all patients are shown. BECTS, benign epilepsy with centro-temporal spikes; ESES, electrical status epilepticus during slow wave sleep; Target Ch, electrode which was selected for slow wave detection; Med, medication*.

### Experimental Protocol and Data Analysis

High-density (hd) sleep EEG combined with auditory stimulation based on real-time slow wave detection was performed during one night in the sleep laboratory. Sleep and wake-up time were scheduled individually according to the subject’s reported habitual bedtime. On the day of the recording night, the entire experimental procedure was explained again in detail to the children and their parents upon arrival at the laboratory. Moreover, the inclusion and exclusion criteria were re-assessed, and the dosage for emergency medication (i.e., Midazolam) was calculated and prepared for the unlikely event of a seizure during the experimental procedure. The regularity of sleep rhythm during the last 7 days, the lack of caffeine consumption, and the absence of convulsive seizures during the 3 days before the recording night were inquired and confirmed by all participants. During the entire sleep recording, the EEG and the child (*via* an infrared camera) were monitored online by an experienced neuropediatrician. Subjective sleep quality during the recording night was assessed by a sleep questionnaire the next morning. Moreover, a follow-up interview by phone was conducted 24–72 h after the recording night asking for the child’s general condition and for any signs of epileptic seizures.

#### High-Density Sleep EEG

For the EEG recording, the electrode net (Electrical Geodesics Sensor Net for long-term monitoring, HydroGel, 128 channels, Electrical Geodesics, Eugene, OR, USA) was adjusted to the vertex and the mastoids, and all electrodes were filled with electrolyte gel to ensure proper data quality throughout the night. Electrooculographic (EOG) and submental electromyographic (EMG) recordings were obtained for visual scoring of sleep stages. For online slow wave detection, two additional electrodes (gold, Grass Technologies, West Warwick, RI, USA) were attached to the earlobes, to serve as reference electrodes (for details see below). Impedances were measured prior to EEG recordings and kept below 50 kΩ in general and below 20 kΩ for those electrodes which were used for the online slow wave detection (i.e., EMG, submental electrodes, electrodes on the earlobes and target electrode). EEG was recorded to the vertex (reference electrode = Cz) and sampled at 500 Hz (0.01–200 Hz).

#### Real-Time Closed-Loop Slow Wave Detection and Acoustic Stimulation

**Figure 1:**
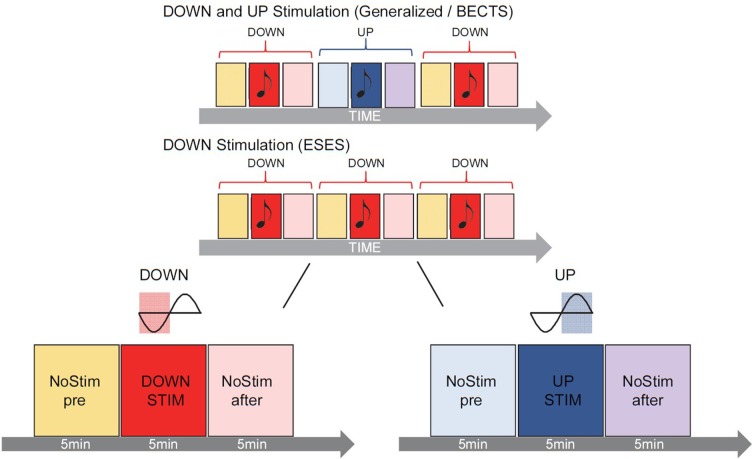
Graphical illustration of the acoustic stimulation. During the night, slow waves were detected online in real time and tones were presented according to a 3 × 5-min-block design. One stimulation cycle consisted of 5 min no stimulation (NoStim_pre_), 5 min stimulation (DOWN or UP) followed by 5 min no stimulation [(NoStim_after_), shown for DOWN (left) and UP stimulation (right) enlarged on the bottom]. In patients diagnosed with benign epilepsy with centro-temporal spikes (BECTS) or generalized spike waves in sleep (*n* = 3) tones onset during the stimulation blocks were played precisely time-locked either to the down-phase (DOWN, reddish color) or up-phase (UP, bluish color) of the detected slow wave, alternately in every other stimulation cycle (top panel). In patients diagnosed with ESES (*n* = 2), only the DOWN stimulation (reddish color) was applied (middle panel). Stimulation was applied throughout the night when subjects were in deep sleep (i.e., N2 and N3, for further details see “Materials and Methods” section).

For real-time slow-wave manipulation during sleep, slow waves were detected online in one specific predetermined electrode (i.e., target electrode). To increase the effect of slow-wave manipulation on the spike-wave activity, the closest electrode to the epileptic focus was selected as the target electrode for each individual patient. If more than one foci were identified, the electrode with the highest amplitude spike-waves (average referenced) was selected. In the patient with generalized spike-waves, we selected the electrode Fz (see Table 1). A similar closed-loop slow wave detection algorithm as reported previously (Fattinger et al., 2017) was applied. In short: a custom LabVIEW (National Instruments, Austin, TX, USA) program was used to detect slow waves of the target electrode in real time (loop time ~15 ms) and to administer acoustic stimuli (50 ms tones, pink 1/*f* noise of ~50 dB, in line with Fattinger et al., 2017) either time-locked to the up-phase (UP, for graphical illustration see Figure 1) or down-phase (DOWN, for graphical illustration see Figure 1) of the detected slow wave. For this purpose, the EEG signal of the target electrode was re-referenced to the mean value of the earlobe electrodes and band-pass filtered (Butterworth 0.5–2 Hz, stopband <0.1 and >10 Hz, stopband attenuation 20 dB, passband attenuation 0.1 dB). Simultaneously, the submental EMG was monitored by continuously calculating the root mean square over 2 s. Anytime the EMG signal reached a certain threshold (continuously adapted by the experimenter), the loop was turned off automatically to prevent sleep disturbance due to acoustic stimulation. During stimulation (i.e., loop turned on) tones were played whenever the EEG signal crossed a default threshold (i.e., 30 μV for DOWN or +50 μV for UP). This threshold was continuously monitored and adapted by the experimenter to allocate tone onset to the minimum or maximum of the slow wave oscillation respectively by using continuous visual feedback of the exact timing of tone onset relative to the ongoing slow waves. Stimulation was only turned on by the experimenter during visually detected stable N2 and N3 sleep (appearance of spindles, K-complexes, and slow waves). Stimulation was applied according to a 3 × 5-min-block design, consisting of 5 min no stimulation (NoStim_pre_), 5 min stimulation (DOWN or UP) followed by 5 min no stimulation (NoStim_after_). One such 3 × 5-min-block was defined as one stimulation cycle and was repeated during N2 and N3 sleep throughout the night (for graphical illustration see Figure 1). If a 5-min-block was interrupted by wake, N1 or REMS for more than 2 min, the stimulation cycle was stopped, and the current stimulation cycle was not considered for the analysis. As soon as the EEG signal showed stable N2 or N3 again, the stimulation algorithm was initiated starting with a new stimulation cycle (i.e., NoStim_pre_). If the interruption was less than 2 min, the stimulation cycle was continued, and the current 5-min-block was prolonged by the time of interruption. Depending on the type of epilepsy of the patient, different stimulation modes were conducted (see Figure 1).

1. Patients diagnosed with BECTS or generalized spike waves in the sleep EEG received DOWN and UP stimulation: Tones were applied either time-locked to the up-phase (5 min stimulation block; UP) or to the down-phase (5 min stimulation block; DOWN): NoStim_pre_—DOWN—NoStim_after_—NoStim_pre_—UP—NoStim_after_—NoStim_pre_—DOWN—NoStim_after_ …
2. Patients diagnosed with ESES received DOWN stimulation only: tones were applied only time-locked to the down-phase (5 min stimulation block; DOWN): NoStim_pre_—DOWN—NoStim_after_—NoStim_pre_—DOWN—NoStim_after_— NoStim_pre_—DOWN—NoStim_after_…

Based on the hypothesis that slow-wave stimulation during the up-phase might increase the level of neuronal synchronization and could, therefore, potentiate spike-wave activity, only the DOWN stimulation mode was chosen for ESES patients. We thus circumvented a saturation effect since ESES patients already show global spike-wave activity during NREM sleep.

#### Sleep EEG Pre-processing

The sleep EEG was band-pass filtered between 0.5 and 40 Hz and down-sampled to 128 Hz. Consecutive sleep stages (i.e., wake, REMS, and NREMS subdivided into N1–N3) were visually scored for 20 s epochs according to standard criteria (Iber et al., 2007), and a visual and semiautomatic artifact removal for all 128 channels was performed (Huber et al., 2000). Only N2 and N3 20 s epochs with no artifacts in any of the 128 channels were considered for further analysis. In line with our previous study (Fattinger et al., 2017), low-SWA (1–2 Hz) of the target electrode was calculated for consecutive 20 s epochs by a Fast-Fourier-Transformation (FFT routine, Hamming window, average of five 4 s epochs) during each 5-min-block separately. To control for general changes in the level of sleep depth across the night, as well as in the course of individual sleep cycles, low-SWA of the target electrode was divided by the mean across all electrodes (i.e., relative low-SWA values). For offline verification of the online slow wave detection algorithm, the instantaneous phase of the EEG signal of the target electrode at tone onset was computed. For this purpose, the EEG signal of the target electrode was preprocessed similar to the online slow wave detection algorithm. The EEG signal was re-referenced to the mean signal from the two earlobe electrodes and band-pass filtered between 0.5 and 2 Hz, using a zero-phase infinite impulse response Butterworth filter (stop-band <0.1 and >10 Hz), to account for filter dependent phase shifts of the EEG signal. Next, a Hilbert transform was computed to obtain instantaneous phase, and phase angles (0° to 360°, subdivided into 12 30°-phase-bins) were defined with 0° corresponding to the negative peak of the slow wave oscillation (for the subdivision of the slow wave oscillation into the 12 different 30° phase-bins see Figure 3). For each stimulation block, the percentage of tones allocated during each 30° phase-bin were calculated.

**Figure 2:**
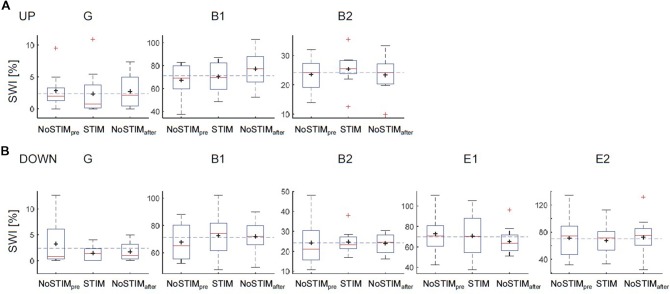
Effects on the spike-wave index (SWI) of the acoustic stimulation during the up-phase **(A)** or the down-phase **(B)** of slow waves for each patient. Boxplot: the middle red line indicates the median, the black cross indicates the mean, the bottom and top of the box indicate the first (Q1 = 25th percentile) and third quartiles (Q3 = 75th percentile), whiskers extend to indicate 1.5 × the IQR (IQR = Q3 − Q1), red crosses correspond to values outside the whisker range. The dashed blue line indicates the mean SWI over all blocks [i.e., for ESES NoSTIM_pre&after_, STIM_down_; for BECTS and generalized spike waves in sleep electroencephalographic (EEG): NoSTIM_pre&after_, STIM_down&UP_]; G, generalized spike waves in sleep EEG; B, BECTS; E, ESES.

**Figure 3:**
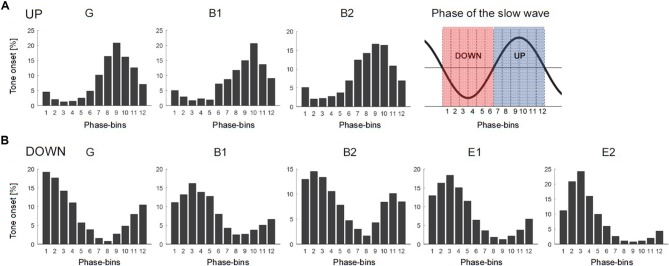
Distribution of tone onset relative to the slow wave oscillation of each subject presented separately for the UP stimulation **(A)** and the DOWN stimulation **(B)**. The phase of the slow wave oscillation was subdivided into 12 30° phase-bins (see graphical illustration in the upper right corner, red: phase-bin 1–6 corresponding to the down-phase, blue: phase-bin 7–12 corresponding to the up-phase). The mean percentage of tone onset allocated during each 30° phase-bin is shown. Note the variability of tone onset allocation across subjects (G, generalized spike waves in sleep EEG; B, BECTS; E, ESES).

#### Assessment of Spike-Wave Index (SWI)

Similar to previous work the density of spike-waves was quantified by a spike wave index (SWI), calculated as the number of visually marked spike-waves divided by the total number of assessed seconds multiplied by 100 (Scheltens-de Boer, 2009). Therefore, for each patient all 5-min-blocks of EEG (i.e., NoStim_pre_, NoStim_after_, DOWN, UP) were cut and encrypted separately with an automatically generated number. SWI was then determined for each block by a well experienced neuropediatric clinician (BS), who was blind to the block condition (i.e., NoStim_pre_, NoStim_after_, DOWN, UP). The following criteria were applied: spike-waves were counted at the electrode of their maximum amplitude. Only spike-waves clearly demarcated from the background activity were considered. EEG phases with relevant artifacts were not assessed. Each 5-min-block was assessed twice and if the number of spikes varied by more than 10% a third and fourth count was performed and the mean of all counts was used for the determination of the SWI.

#### Analysis and Statistics

For each subject, the mean SWI and low-SWA across the night for each stimulation condition (NoStim_pre_, STIM, NoStim_after_) were calculated. Moreover, the SWI and low-SWA was separately compared between NoSTIM and STIM blocks for each stimulation cycle. Thus, for each stimulation cycle, the corresponding STIM block was compared to the NoStim_after_ block and expressed as a percentage of the NoStim_pre_ block to control for baseline level. For each stimulation cycle i:

$$\Delta {\text{SWI}}_{\text{upi}}(\%)=[(SWI_{\text{upi}}-SWI_{\text{afteri}})/SWI_{\text{prei}}]*100$$

$$\Delta {\text{SWI}}_{\text{downi}}(\%)=[(SWI_{\text{downi}}-SWI_{\text{afteri}})/SWI_{\text{prei}}]*100$$

$$\Delta {\text{low-SWA}}_{\text{upi}}(\%)=[({\text{low-SWA}}_{\text{upi}}-{\text{low-SWA}}_{\text{afteri}})/{\text{low-SWA}}_{\text{prei}}]*100$$

$$\Delta {\text{low-SWA}}_{\text{downi}}(\%)=[({\text{low-SWA}}_{\text{downi}}-{\text{low-SWA}}_{\text{afteri}})/{\text{low-SWA}}_{\text{prei}}]*100$$

Note, a positive ΔSWI/Δlow-SWA value indicates that SWI/low-SWA was higher during stimulation, whereas a negative value indicates that SWI/low-SWA was lower during stimulation than after the stimulation block.

For each comparison, all values above or below the mean ± 2 SD were excluded as outliers. All comparisons were graphically explored by box-plots, and the mean over all subjects and data range are presented. Group mean values are presented as mean ± SD. All analyses were performed with the software package MATLAB (Math Works, Version 14a).

## Results

The overall aim of this pilot study was to probe whether acoustic stimulation applied during slow wave sleep is feasible in childhood epilepsy patients. In a second step, we explored whether and how such acoustic stimulation may influence spike-wave activity in relation to the down or the up phase of the ongoing slow waves. To do so, hd EEG was recorded combined with closed-loop real-time slow wave detection, and tones were applied according to a 3 × 5-min-block design. On average, 1,235 ± 155.65 tones were played during 7.67 ± 1.16 DOWN-stimulation-cycles and 1,077.7 ± 112.70 tones during 7.67 ± 0.58 UP-stimulation-cycles (in patients diagnosed with BECTS or generalized spike waves, *n* = 3). In patients diagnosed with ESES, only the DOWN-stimulation-cycles were applied (*n* = 2, 3,360 ± 1,001 tones during 15.5 ± 4.95 DOWN-stimulation-cycles, for details see “Materials and Methods” section and Figure 1).

### Sleep Quality and Clinical Tolerance of Acoustic Stimulation

All patients tolerated the acoustic stimulation well, showing high sleep quality (Tables s 2,3 for objective and subjective sleep quality) and no signs of clinical or non-convulsive electrographic seizures during the recording night. Also in the 24–72 h follow-up assessment, no seizures were reported, and all children were doing well, without any clinical symptoms, indicating that closed-loop acoustic stimulation during slow wave sleep seems to be feasible in these patients.

**Table 2:** Objective sleep quality.

TST (min)	536.13 ± 70.21
NREMS (min)	413.87 ± 42.42
N1 (min)	27.1 ± 12.7
N2 (min)	257.13 ± 50.90
N3 (min)	129.67 ± 25.30
REMS (min)	122.27 ± 31.75
SEF (%)	89.46 ± 8.94
WASO (min)	47.13 ± 44.15
SL (min)	17.4 ± 9.86

*Sleep parameters from visual sleep scoring (mean ± SD; n = 5; NREMS, NREM sleep; N1–N3, NREMS stage 1–3; REMS, REM sleep; TST, total sleep time; SEF, sleep efficiency; WASO, wake after sleep onset; SL, sleep latency)*.

**Table 3:** Subjective sleep quality.

Sleep (0 to 5)		Mood (0 to 5)	
Restless-Quiet	Superficial-Deep	Tired-Recovered	Bad-Good
3.20 ± 0.84	3.60 ± 0.55	3.40 ± 0.89	4.40 ± 0.89

*Subjective rating of sleep quality based on questionnaires. Compared to normal sleep at home sleep quality was rated: Restless = 0, Quiet = 5; Superficial = 0, Deep = 5; and mood after sleep was rated: Tired = 0, Recovered = 5, Bad = 0, Good = 5 (mean ± SD, n = 5)*.

### General Effect of DOWN and UP Stimulation on SWI

In a first step, the mean SWI across the night for each stimulation condition (NoStim_pre_, STIM, NoStim_after_) was computed across all blocks for each individual patient. The SWI showed no systematic change during the DOWN or during the UP stimulation (Figure 2). In a second step, to account for general changes in SWI across the night, the SWI of NoSTIM and STIM blocks were compared for each stimulation cycle separately. This analysis revealed an increase of SWI in three of five patients (mean: 1%, range: −9.6% to 9.4%) during the DOWN stimulation (for individual data points of each patient see red dots in Figure 5), whereas the SWI was reduced after UP stimulation in two of three patients (mean: −19.2%, range: −53.7% to 8.6% (for individual data points of each patient see blue dots in Figure 5).

**Figure 4:**
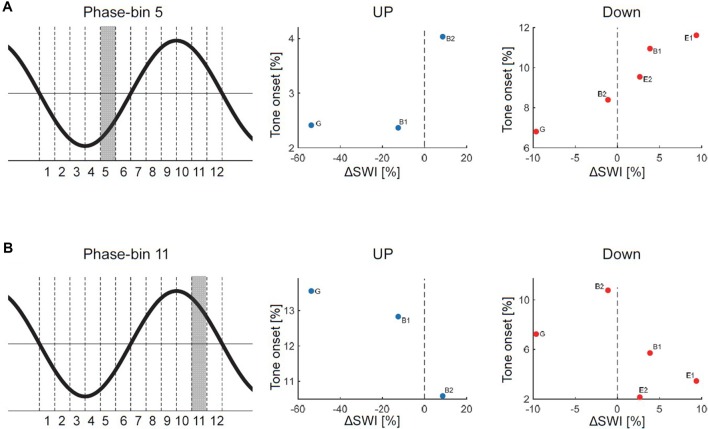
Relationship between phase-timing of tone onset relative to the slow wave oscillation and changes in SWI after stimulation (ΔSWI [%] = (SWI_Stim_ − SWI_after_)/SWI_pre_)* 100. A positive ΔSWI value indicates that SWI was higher during stimulation, whereas a negative value indicates that SWI was lower during stimulation than after the stimulation block). The phase of the slow wave oscillation was subdivided into 12 30° phase-bins (see graphical illustration). The mean percentage of tone onsets allocated during each 30° phase-bin was calculated. **(A)** Data points for each patient are shown for the UP and DOWN stimulation separately for the 5th phase-bin (i.e., the onset of the EEG positive trend). The distribution of the individual data points might indicate that the more tones were played during the 5th phase-bin, the more the SWI was increased during stimulation. **(B)** Data points for each patient are shown for the UP and DOWN stimulation separately for the 11th phase-bin (i.e., the onset of the EEG negative trend). The distribution of the individual data points might indicate that the more tones were played during the 11th phase-bin the more the SWI was reduced during stimulation (G, generalized spike waves in sleep EEG; B, BECTS; E, ESES).

**Figure 5:**
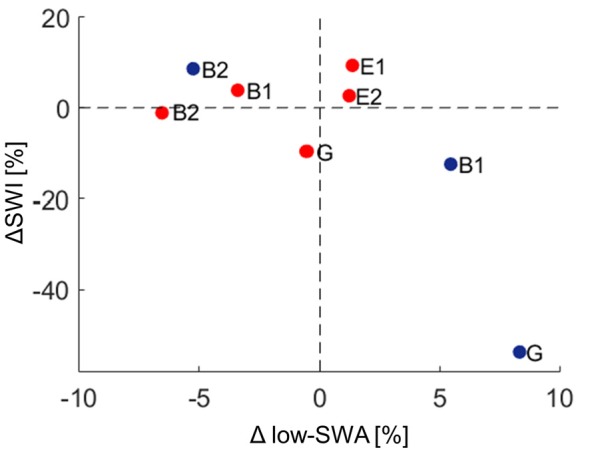
Relationship between spike-wave index (ΔSWI) and Δlow-slow wave activity (low-SWA) for DOWN (red dots) and UP (blue dots) stimulation pooled. Data points for each patient are shown for the UP and DOWN stimulation separately (ΔSWI [%] = (SWI_Stim_ − SWI_after_)/SWI_pre_)* 100; Δlow-SWA [%] = (low-SWA_after_ − low-SWA_STIM_)/low-SWA_pre_)* 100. A positive ΔSWI/Δlow-SWA value indicates that SWI/low-SWA was higher during stimulation, whereas a negative value indicates that SWI/low-SWA was lower during stimulation than after the stimulation block. G, generalized spike waves in sleep EEG; B, BECTS; E, ESES.

### Exact Slow Wave Phase-Timing of Acoustic Stimulation

The effect of acoustic stimulation on spike-wave activity may depend on the phase-timing of tone onset relative to the ongoing slow waves. Thus, in a next step, we assessed the exact phase-timing of the acoustic stimuli relative to the ongoing slow waves for each patient separately. For this purpose, we computed offline the instantaneous phase of the EEG signal of the target electrode at tone onset (for details see “Materials and Methods” section). For both kinds of stimulation (i.e., DOWN and UP) we observed a variable phase-timing of tone onsets relative to the slow wave oscillation across patients (see Figure 3 for the distribution of tone onsets relative to the slow wave oscillation of each subject separately). Thus, this finding indicates that with our close-loop auditory stimulation tool, tones were distributed over a rather broad phase-range during the DOWN or UP stimulation across patients. To exemplify the relationship between phase and potential systematic effects on SWI, we calculated the number of tone onsets for each phase-bin and plotted them against the change in SWI. This explorative analyses revealed promising though non-significant trends for phase-bin 5 (i.e., just after the onset of the EEG positive trend) and phase-bin 11 (just after the onset of the EEG negative trend; Figure 4).

### Effect of Acoustic Stimulation on Low-SWA

In a final step, the effect of acoustic stimulation on low-SWA was analyzed. Thus, changes in low-SWA were calculated for each stimulation-cycle separately (see “Materials and Methods” section). As found for ΔSWI, the outcome of Δlow-SWA due to acoustic stimulation was variable across patients. Tones time-locked to the down phase reduced low-SWA in 3/5 patients (mean: −1.6%, range: −6.6% to 1.4%), whereas tones time-locked to the up phase increased low-SWA in 2/3 patients (mean: 2.9%, range: −5.3% to 8.3%). However, this variability of Δlow-SWA seemed not to be explained by the variability of tone onsets relative to the slow wave oscillation (no relationship between Δlow-SWA and phase-timing of tone onset was observed). Moreover, no clear relationship between Δlow-SWA and ΔSWI was observed (Figure 5).

## Discussion

In this pilot study, we investigated the effect of acoustic stimulation time-locked to sleep slow waves on spike-wave activity in childhood epilepsies with activation of spike-wave activity during sleep. We observed a high sleep efficiency in all patients and no signs of clinical or non-convulsive electrographic seizures during the recording night. This indicates that acoustic stimulation during sleep was well tolerated in all patients. However, our block design did not result in a consistent effect of DOWN and UP stimulation on SWI.

Different factors of our experimental approach might have contributed to this lack of a systematical, phase-dependent effect of auditory stimulation on SWI, which will be discussed below. First, offline quantification of tone onset phase-timing relative to the ongoing slow waves during the DOWN and UP stimulation revealed that tones were applied over a broad phase-range across patients (Figure 3). Thus, our closed-loop auditory stimulation tool was not sufficiently accurate in targeting the same phase of the ongoing slow waves in different patients. This variability may have contributed to the observed variable effects of acoustic stimulation of spike-wave activity across patients. Another major limitation of the current pilot study was the block-design of acoustic stimulation. This procedure was selected to explore the different effects of up and down stimulation on SWI within the same patient and the same night to reduce the burden for the patients and their families (i.e., no multiple night recordings for the same patient). However, as a consequence, the direct comparison of SWI before, during and after stimulation is difficult, because of a biased distribution of the different blocks of a stimulation cycle (i.e., NoSTIM_pre_—STIM—NoSTIM_after_) within sleep episodes. More specifically, the NoSTIM_pre_ blocks tended to be at the beginning of a deep sleep episode. Moreover, because SWA is building up in the course of a sleep episode, SWA tended to increase in the course of a stimulation cycle. This evolution of SWA within the stimulation cycle is likely affecting the SWI. In line with others (Boly et al., 2017), we observed a strong relationship between absolute SWI and SWA in all our patients: the higher the SWA, the higher the SWI (data not shown). Another limitation of our block design is related to the well-known homeostatic regulation of slow waves (Borbély and Achermann, 2011). Thus, stimulation-induced changes in SWA might have resulted in corresponding rebound effects of SWA during subsequent NoStim epochs. Hence, in an exploratory analysis, the SWI of NoSTIM and STIM blocks were compared for each stimulation cycle separately to account for such general changes in SWI across the night (see “Materials and Methods” section). As shown in Figure 5, during DOWN stimulation the SWI was increased in three of five patients, whereas the SWI was reduced after UP stimulation in two of three patients (each patient is represented by a circle in the scatterplots). Yet, to further elaborate on the relationship between the phase-timing of acoustic stimuli and the changes in SWI, the number of tone onsets for each phase-bin were plotted against the observed changes in SWI of each patient. Interestingly, for phase-bin 5 (i.e., just after the onset of the EEG positive trend), the distribution of the individual data points may reveal some first evidence that the more tones were applied during this phase-bin 5, the more the SWI was increased during stimulation. On the other hand, the more tones were applied during phase-bin 11 (i.e., the onset of the EEG negative trend) the more the SWI was reduced during stimulation (Figure 4). Thus, based on these preliminary results, we postulate that improved phase timing, e.g., as proposed by Santostasi et al. (2016), may significantly increase the effect of acoustic stimulation on SWI. However, the low number of subjects did not allow any statistical analyses of our data and thus, these findings remain to be replicated in larger samples of patients.

The effect of acoustic stimulation on slow waves itself has been experimentally investigated in healthy humans. SWA enhancement by acoustic stimulation during the up-phase has been reproducibly shown, in young and elderly (Ngo et al., 2013; Santostasi et al., 2016; Leminen et al., 2017; Papalambros et al., 2017). While stimulation time-locked to the up-phase increases SWA, tones applied during the down-phase induce the opposite effect, i.e., decreasing SWA (Ngo et al., 2013; Fattinger et al., 2017). These effects were most pronounced in the low-SWA frequency range (Fattinger et al., 2017). In the present experiment, similar to the changes in SWI, the low number of subjects did yield inconclusive effects of acoustic stimulation on low-SWA. More specifically, though in the majority of patients acoustic stimulation-induced effects in the expected direction (i.e., in three out of five patients low-SWA was reduced during down stimulation and in two out of three patients low-SWA was increased during up stimulation), no statistics can be provided for this result. Moreover, with our data, exploratory analysis revealed no clear relationship between these changes in low-SWA and ΔSWI (Figure 5).

Even though the primary outcome of our study was negative, we have good reasons for further exploration of novel methods allowing a systematic manipulation of spike-waves. There is growing evidence for adverse consequences of interictal spike-wave activity on cerebral functioning particularly during brain development (for review, see Sánchez Fernández et al., 2015). For instance, in ESES patients the characteristic developmental deterioration has been linked to impaired slow-wave-related changes in network synchronization in the course of a night (Bölsterli et al., 2011; Urbain et al., 2011; Bölsterli Heinzle et al., 2014). The underlying pathomechanism is, however, unknown. Influencing spike wave generation by acoustic stimulation may offer such new insights in the understanding of the emergence of spike waves and the associated cognitive deficits. These insights may facilitate new treatment strategies for epilepsy and cognitive deficits, beyond pharmacological treatment. This is crucial not only for drug-resistant epilepsies and patients with intolerable side effects of anticonvulsants but also for patients with less severe epilepsies presenting with specific cognitive deficits (i.e., Rolandic epilepsy). Moreover, also other brain disorders like ADHD (e.g., Kanazawa, 2014), and even Alzheimer’s (Lam et al., 2017) were recently associated with increased occurrence of spike-wave activity during sleep. Future studies should explore whether closed-loop acoustic stimulation during sleep may represent a novel treatment option for these patient groups.

## Data Availability

The ethical approval granted to the authors by the IRB does not allow the publication of the raw data online. If readers would like to re-analyze the dataset (for different purposes), additional ethical approval (on an individual user and purpose basis) will be required. The authors would be happy to support additional ethical approval applications from researchers for access to this dataset. The datasets generated for this study are available on request to the corresponding author.

## Ethics Statement

The study was approved by the Ethics commission of the Kanton Zurich. All participants and their parents were informed about the study procedure and gave consent for their participation.

## Author Contributions

SF, BS, and RH conceived the experiment. SF, BH, GR, LA, and BS performed the measurements, and SF analyzed the data. SF, BS, and RH wrote the manuscript. All authors contributed to the scientific discussion and manuscript revisions.

## Conflict of Interest Statement

The authors declare that the research was conducted in the absence of any commercial or financial relationships that could be construed as a potential conflict of interest.
